# COVID‐19 and the impact on gynecologic cancer care

**DOI:** 10.1002/ijgo.13868

**Published:** 2021-10-20

**Authors:** Ranjit Manchanda, Samuel Oxley, Sadaf Ghaem‐Maghami, Sudha Sundar

**Affiliations:** ^1^ Wolfson Institute of Preventive Medicine Cancer Research UK Barts Centre Queen Mary University of London London UK; ^2^ Department of Gynecological Oncology St Bartholomew's Hospital Barts Health NHS Trust London UK; ^3^ Faculty of Public Health and Policy Department of Health Services Research London School of Hygiene and Tropical Medicine London UK; ^4^ Faculty of Medicine Department of Surgery and Cancer Imperial College London London UK; ^5^ Institute of Cancer and Genomic Sciences University of Birmingham Birmingham UK

**Keywords:** cancer care, COVID‐19, FIGO Cancer Report, gynecology, oncology

## Abstract

The COVID‐19 pandemic resulted in significant reconfiguration of gynecologic cancer services and care pathways across the globe, with a transformation of working practices. Services had to adapt to protect their vulnerable patients from infection, whilst providing care despite reduced resources/capacity and staffing. The international gynecologic cancer community introduced modified clinical care guidelines. Remote working, reduced hospital visiting, routine COVID‐testing, and use of COVID‐free surgical areas/hubs enabled the ongoing and safe delivery of complex cancer care, with priority levels for cancer treatments established to guide decision‐making by multidisciplinary tumor boards. Some 2.3 million cancer surgeries were delayed or cancelled during the first peak, with many patients reporting significant anxiety/concern for cancer progression and COVID infection. Although COVID trials were prioritized, recruitment to other cancer trials/research activity was significantly reduced. The impact of resultant protocol deviations on outcomes remains to be established. During the recovery healthcare services must maintain capacity and flexibility to manage future surges of infection, address the large backlog of patients with altered or delayed treatments, along with salvaging screening and prevention services. Training needs/mental well‐being of trainees need addressing and staff burnout prevented. Future research needs to fully evaluate the impact of COVID‐19 on long‐term patient outcomes.

## Synopsis

This article reviews the global changes introduced to gynecologic cancer care due to COVID‐19, and their impact on services and patients. Key lessons are considered for the recovery and future.

## 1 COVID‐19 AND THE IMPACT ON GYNECOLOGIC CANCER CARE

Coronavirus disease 2019 (COVID‐19) is caused by the severe acute respiratory syndrome coronavirus 2 (SARS‐CoV‐2). The World Health Organization declared COVID‐19 a pandemic in March 2020. The ongoing pandemic has led to a global crisis disrupting most health systems and economies worldwide, leading to at least 195 million confirmed infections with 4 million deaths, as of July 2021.^1^ It has had a profound effect on many areas of health care and a major impact on cancer care. The impact of this pandemic has been felt differently across the globe due to large variations in seroprevalence, varying disease burden, existing health infrastructure, and available healthcare capacity and resources. An overall decrease in life expectancy^2^ as well as a significant increase in all‐cause mortality has recently been observed in some countries, with higher levels of excess deaths occurring in nonwhite populations.^3^

All countries took multiple steps to reduce transmission of COVID‐19, including public lockdowns, self‐isolation for those with the disease and their contacts, contact tracing, and shielding of individuals with high‐risk pre‐existing medical conditions. Areas of high prevalence saw significant pressures on hospital inpatient beds including intensive care units (ITU), as well as staffing levels.^4^ It led to reallocation of resources and an increase in hospital beds and ITU capacity for COVID‐19 patients at the expense of both elective nonurgent patient care activity and urgent care activity including oncology services. Health systems and gynecologic cancer services have had to cope with a number of additional factors/stresses, including staff sickness and self‐isolation, staff redeployment for COVID care, reduced theatre availability/capacity for elective oncology, reduced ITU access for complex surgery, reduced palliative care access, supply chain shortages (including personal protective equipment, PPE), reduced hospital visits, and moves toward remote consultations.^5^, ^6^ In the initial period, some patients refused surgery due to concerns around contracting COVID‐19 and there was a fall in the number of patients seeking care via their general practitioners or emergency settings.

COVID‐19 has a highly variable clinical presentation with approximately 44% of transmissions occurring in the presymptomatic stage,^7^ while many carriers may never show symptoms^8^, ^9^ but can transmit the virus to others.^10^, ^11^ The majority of patients suffer no or mild upper respiratory tract symptoms,^11^ whilst a minority are at highest risk of severe lower respiratory tract infection and need hospitalization. Mortality occurs predominantly through respiratory failure; however, sepsis, thromboembolism, and acute renal and multiorgan failure contribute to this complex clinical entity, requiring prolonged stays on ITUs.^12^

Early in the pandemic, cancer patients were identified as being at higher risk for COVID infection, increased morbidity, ITU care, and mortality.^13^, ^14^, ^15^ Other predictors of severe disease and mortality included age, comorbidities such as obesity, diabetes, asthma, other medical morbidities, and black/Asian ethnicity.^15^ The gynecologic cancer patient population was thus considered vulnerable, with overlapping risk factors and immunosuppression arising from cancer and its treatments. An international study of 1128 patients undergoing surgery across all surgical disciplines found 30‐day mortality rates in patients who develop COVID‐19 in the perioperative period to be 19% following elective surgery, 26% following emergency surgery, and 27% following cancer surgery.^16^, ^17^ Gynecologic cancer services faced the dual challenge of continuing to provide oncological care often with capacity constraints, whilst protecting their vulnerable patients from the risk of COVID exposure and its sequelae. Thus, consideration was initially given to restricting surgery or chemotherapy to reduce COVID risk to patients. However, these initial concerns were not born out as subsequent evidence demonstrated such treatments did not increase the risk of hospitalization or death outside the perioperative period or during cytotoxic chemotherapy, beyond those of previously identified risk from age or comorbidities.^4^, ^18^

The unprecedented reallocation of healthcare services necessitated by the pandemic across high‐, middle‐, and low‐income countries,^19^ coupled with staff shortages and capacity constraints, forced an urgent re‐evaluation of the clinical justification for each aspect of treatment, weighing oncological benefit against available resources. In terms of ethical principles, justice and nonmaleficence required due consideration alongside beneficence, autonomy, and equity, as clinicians learnt to practice in this unfamiliar situation.^20^ Modified clinical guidelines for cancer care became necessary to provide a systematic, equitable, and evidence‐led framework during the pandemic, given the adaptation of or reduction in usual services. Deviations from usual standard of care may be appropriate in the context of what can be safely delivered during the pandemic. The international gynecologic oncology community developed modifications to clinical care across the spectrum of surgery, chemotherapy, radiotherapy, and treatment timelines from first presentation to relapse and palliation. National healthcare organizations in France, the UK, USA, Italy, and Australia issued guidance for general oncology services from March 2020, shortly followed by gynecologic cancer specific statements from organizations in the UK, USA, Europe, Canada, Asia, Australia, and New Zealand.^21^ Pragmatic modifications to the gold standard of care were suggested according to availability of resources and clinical services, based primarily on expert opinion and review of pre‐existing evidence of benefit. Initial considerations were given to reducing the number of surgical procedures associated with prolonged operative time, risk of major blood loss, ITU admission, or increased infection risk to staff. A global modelling analysis suggested that around 38% of cancer operations and 82% of benign surgical procedures may be postponed during the pandemic.^22^ Other analysis highlighted the significant impact on survival even modest delays in surgery may incur on cancer outcomes.^23^ The need for care of cancer patients to be prioritized over patients with benign diseases was universally recognized. Additionally, the need to tackle/minimize diagnostic delays occurring during the pandemic became apparent to prevent significant additional avoidable mortality.^24^

## 2 ADAPTATIONS TO CLINICAL SERVICES AND PATHWAYS

There has been significant service reconfiguration, transformation of working practices, and changes to cancer care delivery pathways as a result of the COVID‐19 pandemic.^25^ An important reason was to reduce the risk of infection to patients and staff, from other patients, staff members, and visitors. Key steps included reduction in people attending a given clinical site, along with use of handwashing, face masks, public distancing, PPE, and safe hygiene practices. Staff struggled with PPE shortages in both low‐ and high‐income countries—an issue leaving many staff at higher risk of infection.^26^ Visiting was reduced for all hospital attendances for inpatient and outpatient care. It was restricted to new patients or essential consultations for acute oncologic issues or those undergoing active treatment and the most vulnerable patients.^19^ Attendance of family members was restricted. Remote or telemedicine/telephone outpatient consultations were undertaken to reduce hospital attendance. Routine clinics such as regular follow‐up and preassessment were conducted remotely. The use of patient‐initiated follow‐up was advocated where appropriate. Patients who required definite examination or breaking of bad news were advised to attend in person and benefitted from support from specialist nurses as usual. Multidisciplinary team or tumor board meetings were conducted remotely or, where not possible, sufficient distancing between staff was ensured to reduce the risk of staff transmission. As testing capacity became established, universal testing of patients prior to and upon admission was implemented. Preoperative COVID‐19 testing and self‐isolation protocols (e.g. 14‐day self‐isolation and COVID‐19 swab 72 hours before surgery) were introduced prior to gynecologic cancer surgery. Additionally, over time, surveillance testing of healthcare staff was introduced and has become a key tool in reducing transmission within some hospitals.^27^ The use of “COVID‐free” areas within hospitals or COVID‐free hospitals/surgical hubs with separate staff enabled surgical and nonsurgical cancer services to continue. This approach of establishing COVID‐19‐free surgical pathways with segregation of the operating theater, ITU, and inpatient ward areas was shown to reduce postoperative SARS‐CoV‐2 infection and postoperative pulmonary complication rates.^28^ In some centers, surgery was allocated through centralized triage and decision‐making based on newly established national guidelines.^29^ The introduction of vaccination from December 2020 for staff and more recently for patients is a key step forward in minimizing infections and reducing treatment morbidity.

## 3 ADAPTATIONS TO DIAGNOSTIC SERVICES

Changes to diagnostic pathways aimed to simplify the process, reducing hospital attendance and demand on clinical time, whilst still providing a safe process for patients. Greater flexibility was incorporated into triaging suspected cancer referrals from primary care. Examples of changes introduced include:

- Telephone or virtual assessment without the need for clinical examination followed by direct investigation with hysteroscopy and ultrasound in women with postmenopausal bleeding.
- Maximizing out‐patient hysteroscopy due to the reduced availability of operating theatres.
- Insertion of the levonorgestrel‐releasing intrauterine system (LNG‐IUS) at the time of initial hysteroscopy in case of abnormal findings, to minimize face‐to‐face visits and treatment delays where surgical treatment is constrained.
- One‐stop clinics preferred over multiple visits.
- Patient‐initiated follow‐up to ensure those with multiple episodes of bleeding could attend for clinical examination when required.
- Evaluation of adnexal masses using ultrasound or MRI and established rules for triage, e.g. International Ovarian Tumour Analysis or the Risk of Malignancy Index (RMI) etc. In cases of low index of suspicion for malignancy (e.g. RMI <200), surgery could be deferred for 3–6 months, with virtual follow‐up.
- For those requiring assessment in secondary care, such as patients with postmenopausal bleeding, telephone or virtual assessment followed by direct investigation with hysteroscopy and ultrasound is preferred without the need for clinical examination if possible (given a normal cervical smear history).
- For those with suspected ovarian cancer, using cytology to guide treatment decisions if there was inadequate or restricted access to usual image‐guided biopsies.

## 4 ADAPTATIONS TO TREATMENT

The disruption to cancer services and capacity constraints have required prioritization of treatment for those patients who are most in need. Three priority levels were recommended by the British Gynaecological Cancer Society (BGCS) and UK National Health Service (NHS) to determine the timescales required for surgical treatments during the pandemic (Table 1).^29^ Patients are advised of the risks of perioperative COVID‐19 infection as well as the risk of reduced survival with delayed treatment. It is important that any delays or changes to gold standard treatment should be recorded, communicated clearly with patients, and made with multidisciplinary input. Patients whose treatment is deferred should be tracked. The BGCS has devised a harms template that can be used.^29^ Considerations were made to improve the safety of procedures undertaken.^30^ COVID‐19 testing should occur prior to surgery; where a patient tests positive their treatment should be delayed by 2–4 weeks to allow recovery owing to the heightened morbidity from perioperative COVID‐19 infection. Vaccination for COVID‐19 significantly reduces the risks of infection and is recommended for all women planned for and undergoing cancer treatment.^31^, ^32^, ^33^

**TABLE 1:** Priority levels for surgery^a^

Priority	Surgery	Examples
Level 1a	Emergency: operation needed within 24 h to save life	Anastomotic leak, bowel perforation, peritonitis, burst abdomen, torsion or rupture of suspected malignant pelvic masses, heavy bleeding from molar pregnancy
Level 1b	Urgent: operation needed within 72 h	Acute mechanical intestinal obstruction/impending perforation, life‐threatening bleeding from cervical or uterine cancer
Level 2	Elective surgery with expectations to cure, performed within 4 weeks to save life/progression of disease beyond operability Additional prioritization based on urgency of symptoms, complications (such as local compressive symptoms), biological priority (expected growth rate) of individual cancers	Suspected germ cell tumors, intrauterine brachytherapy for cervical cancer, pelvic masses suspicious of ovarian cancer, early‐stage cervical cancer, high‐grade/high‐risk uterine cancer, debulking surgery (timed to chemotherapy schedules) for advanced epithelial ovarian cancer where ITU/HDU capacity permits
Level 3	Surgery can be delayed by 10–12 weeks with no predicted negative outcome	Where risk to the patient from surgery during the pandemic outweighs benefit Early‐stage, low‐grade uterine cancer (treated with LNG‐IUS/oral progestogens), low volume cervical cancer completely excised at loop excision Surgical resection of slow‐growing recurrences of ovarian, endometrial, and cervical cancer postponed or alternatively managed with chemotherapy or radiotherapy, particularly in the absence of proven survival benefit for secondary debulking

^a^ Source: British Gynaecological Cancer Society.^29^

Routine primary or interval debulking surgery for advanced ovarian cancer was delayed at the start of the pandemic, with neoadjuvant chemotherapy given due to initial concerns regarding surgical morbidity as well as the increased resource requirements, including intensive care needs for complex debulking surgery and existing capacity constraints. Patients with recurrence needing secondary debulking were classed as lower surgical priority and managed with chemotherapy.

Measures promoted to minimize the duration of hospital stay included enhanced recovery pathways and the use of laparoscopy where possible. Available data suggested that the risk of SARS‐COV‐2/COVID‐19 transmission to staff from laparoscopic surgery for gynecologic procedures is low and hence minimal access surgery can be continued during the pandemic.^29^, ^34^ However, reasonable modifications to technique including minimization leakage from evacuation of smoke or carbon dioxide from the abdomen, and the use of FFP3/N95 face masks by staff should be implemented. Where surgery may involve the gastrointestinal tract—an area known to carry virus particles—consideration should be given to laparotomy.

National and international guidelines covered the use of chemotherapy and modifications to medical oncology practices, including prioritization levels for treatment. Prior to starting treatment, the Cockroft–Gault or Wright methods were recommended to calculate glomerular filtration rate rather than the use of radionucleotides.^29^ Cancer networks needed to consider the impact on the supply of medications and work with regional and national organizations to minimize disruptions. The National Institute of Health and Care Excellence (NICE) suggested six levels of priority for chemotherapy (Table 2)^35^ and five priority levels for radiotherapy treatment.^36^

**TABLE 2:** Priority levels for chemotherapy^a^

Priority	Treatment	Examples
Level 1	Curative therapy with a high (>50%) chance of success	Chemotherapy for germ cell and gestational trophoblastic tumors. Concurrent chemoradiation for cervical cancer
Level 2	Curative therapy with an intermediate (20%–50%) chance of success	Chemotherapy for women with high‐grade serous or endometrioid ovarian cancer, including those with extrapelvic ovarian cancer. Maintenance bevacizumab was discouraged, maintenance with PARP inhibitors was promoted for BRCA patients
Level 3	Curative therapy or adjuvant therapy with 10%–20% chance of success, or noncurative treatment with a >50% chance of 1‐year survival prolongation	Platinum sensitive relapse; advanced, high‐grade endometrial cancer; however, endocrine treatment may be an appropriate alternative for many other endometrial cases
Level 4	Curative therapy with a low (0%–10%) chance of success. Noncurative therapy with an intermediate (15%–50%) chance of more than 1‐year life extension	Chemotherapy for first recurrence of cervical and endometrial cancer (good performance status), or advanced previously untreated disease. Some women with platinum‐sensitive relapsed ovarian cancer
Level 5	Noncurative therapy with a high (more than 50%) chance of palliation/temporary tumor control but less than 1‐year life extension	Chemotherapy for platinum‐resistant ovarian cancer and recurrent endometrial cancer
Level 6	Noncurative therapy with an intermediate (15%–50%) chance of palliation/temporary tumor control and <1‐year life extension	Chemotherapy for metastatic or recurrent cervical cancer or endometrial cancer in second recurrence

^a^ Source: NICE.^35^

Radiotherapy guidelines^29^, ^36^ included the use of hypofractionated schedules to provide equivalent dose with fewer hospital attendances, and simplifications of technique. High priority level 1 treatments included radical radiotherapy for cervical, vaginal, and vulvar cancers. Level 2 treatments included urgent palliative radiotherapy to preserve function such as in malignant spinal cord compression and palliative radiotherapy to stop bleeding. Low priority cases include palliative radiotherapy for symptom control, and adjuvant radiotherapy for endometrial cancer.

## 5 IMPACT ON SURGERY

The COVID‐19 pandemic has severely disrupted cancer care across the whole spectrum of prevention, diagnosis, surgery, oncology treatments, and palliative care. Modelling has estimated that approximately 2.3 million cancer surgeries will have been delayed or cancelled during the pandemic's first peak (March–May 2020).^22^, ^37^ The impact on cancer diagnostics is also estimated to be substantial, with more than 350 000 fewer people than usual being referred for a rapid referral for suspected cancer in the UK between March and September 2020, largely owing to fewer people seeking primary care advice.^38^ The uptake of screening programs has been reduced and elective preventive surgery has been delayed.

The COVIDSurg gynaecological cancer study investigating outcomes in first‐line management of women with gynecologic cancer reports that at least 15% of women with gynecologic cancer have suffered disruption/change to usual first‐line surgery.^39^ Current data show that major morbidity and mortality from gynecologic cancer surgery in patients selected to undergo surgery during the pandemic is comparable to pre‐COVID times.^28^ Studies have also clearly shown that introduction of safe COVID‐free pathways and establishing cancer care in COVID‐free elective care hospitals that do not provide care for COVID‐19 patients ensure that cancer care can safely continue even during the pandemic. Nevertheless, cancer surgery has been severely tested and the resilience of the healthcare system to provide cancer care must be boosted by investment from both government and private sector players worldwide.

Prioritization frameworks issued by the BGCS and NICE (Tables 1 and 2), as well as other international societies, have attempted to balance the risk from treatment in COVID‐19 exposed environments and the availability of resources, including intensive care beds, against the impact of such delays on oncological outcomes. Long‐term data on the impact of such prioritization frameworks are awaited from the UKCOGS study (www.ukcogs.org.uk).

## 6 IMPACT ON PATIENTS

Fifty‐four percent of women with ovarian cancer report that their treatment has been impacted due to the COVID‐19 pandemic, and 27% of women could not access care as they did before the pandemic. A patient experience survey conducted by the charity Cancer Research UK investigating the overall impact of care reported that gynecologic cancers were among the most affected, with 78% patients with gynecologic cancers reporting an impact.^40^ Questionnaire surveys investigating patient perceptions show that fear about COVID‐19 and anxiety about cancer progression due to a change in cancer care during COVID‐19 is a frequent and serious concern for cancer patients.^41^

It is important to understand that healthcare givers and patients/carers can perceive the impact of change very differently. Changes in care such as telephone follow‐up rather than face‐to‐face follow‐up, earlier discharge from hospital, and stopping visiting may all be seen by healthcare providers as relatively minor changes to overall outcome. However, these are seen as major changes to usual care by patients. It is difficult to quantify the impact these changes may make on the relationship of trust between doctor and patient/carer. Clinical staff too are constrained by their own psychological response to risks brought on by the pandemic, including the ongoing risk of becoming infected with COVID‐19, which placed them in an uncertain position. Healthcare staff are also vulnerable to the impact of COVID‐19; it is vital that this is recognized and burnout is prevented.

Patients also seek information amongst themselves and conduct lay risk assessments through online patient groups; for instance, regarding what they can or cannot do during periods when they were advised to shield from COVID‐19 exposure.^42^ Additionally, the impact of change to planned cancer care, as perceived by patients, is a source of huge distress—patients and carers worry about the long‐term impact on their trajectory, and it is interesting to reflect that patients perceive their care plan to be the only or optimal one. Findings are consistent with those identified from questionnaire surveys of patients in the published literature.^41^ Patients struggle with the risk calculus of individualized lay risk assessment in non‐COVID times and this struggle is embellished by the uncertainty of COVID‐19. It is important to improve patient communication and education, as well as develop better supportive care strategies. Cancer charities have also served as the first port of call for patients during the pandemic, providing guidance and support. Furthermore, healthcare providers working in collaboration with reputed cancer charities can establish innovative ways of supporting patients with robust evidence and guidance. The European Society of Gynaecological Oncology (ESGO) has instituted, together with ENGAGe, a series of COVID‐19 webinars for patients. In the UK, the BGCS has worked with national gynecologic cancer charities to address patient concerns through the pandemic proactively (https://www.bgcs.org.uk/public‐information/covid‐19/).

## 7 IMPACT ON CLINICAL TRIAL MANAGEMENT

COVID‐19 has had a huge impact on clinical trial recruitment, management, and research activity. Trial delivery and research teams had to deal with staff shortages, sickness, redeployment to COVID‐19 care, implementation of remote working practices, and reduction in research funding. Laboratory services were reallocated to expand COVID‐19 testing services or significantly reduced as universities were shut. New trial set‐up and recruitment including to ongoing trials was paused, with a 65%–79% drop in recruitment reported by some.^43^ However, COVID‐19‐related trials/research were prioritized along with management of patients already recruited to clinical trials. Mitigation strategies were introduced for ongoing trials and patient management, including remote assessments, reduced clinical visits, etc. The impact of protocol deviations on trial outcomes remains to be established.

## 8 IMPACT ON SURGICAL TRAINING

A recent study showed that COVID‐19 has had a negative impact on surgical gynecologic oncology training.^44^ Twenty‐eight percent of trainees had COVID‐19 and this was associated with increased anxiety/depression. Trainees had to deal with redeployment, lack of PPE, reduction in household income, reduced surgical exposure, and negative impact on psychological/mental well‐being. While departmental teaching continues, the frequency was reduced and it was conducted virtually. The recovery phase needs to address lost training opportunities and also focus on improving the mental well‐being of trainees.

## 9 MOVING FORWARD

With different countries at different stages in the implementation of vaccination programs, alongside repeating infection peaks, there is a need to plan for further epidemics of COVID‐19, COVID‐19 variants, and other infectious diseases. It is necessary to evaluate those measures that have been successful and brought benefits that remain outside of the context of a pandemic, and those that have brought difficulties. Some measures introduced have seen an acceleration of pre‐existing trends (and are therefore more likely to persevere), whilst others have been forced in adversity. Whilst every cancer center and network will reflect on their own ways of working, and every infectious disease and setting will bring unique challenges, we offer some considerations to guide planning. Key considerations include flexibility in service provision and preparedness for increased capacity to allow a rapid response to future surges of infection. Extra resources will be required to enable the recovery of diagnostic and treatments for both cancer and precancerous treatments, as well as the very high global burden of delayed benign disease. Modifications to patient pathways need to be evaluated, with a return to standard‐of‐care treatments where possible. Guidance has been published on the management of women who have been treated via nonstandard pathways, including for example those reconsidering cytoreductive surgery for patients with ovarian cancer who were not adequately treated, and surveillance imaging for those with high‐risk endometrial cancer who did not receive adjuvant radiotherapy.^45^ Cancer screening and prevention services need a fresh impetus to minimize long‐term detriment. Certain measures introduced during the pandemic may bring long‐term benefits in the right contexts, for example, including greater use of one‐stop clinics and virtual triage prior to investigations, and should be continued if they are seen to be efficient and cost‐effective. Remote meetings and educational activities have the potential to share learning across wider networks and should be continually developed.

Important messages:

- It is possible to operate safely on gynecologic cancer patients during the COVID‐19 pandemic, with precautions.
- Hospitals and health systems should have resilient elective care pathways so that cancer is managed in safe areas, away from where patients with COVID‐19 are being looked after.
- It is critical to ensure COVID‐19 vaccination for all cancer patients.
- Counter misinformation by working with cancer charities and patient support groups, developing a clear communications plan to reach patients.
- It is important to focus on salvaging screening and prevention services as this will have long‐term impact, especially for cervical cancer.
- We must plan for the recovery and backlog of patients whose treatments have been delayed or altered.
- We need to prepare for additional surges or outbreaks of infection.
- Steps need to be taken to minimize staff burnout and attrition.
- Trainees need additional support that addresses both their training needs as well as mental well‐being.
- Research is needed to evaluate the impact of COVID‐19 on changes to gynecologic cancer patient care and long‐term patient outcomes.

## AUTHOR CONTRIBUTIONS

All authors contributed to manuscript writing, review, and approval.

## CONFLICTS OF INTEREST

Relating to the submitted work, RM reports grant funding received from BGCS to support work into COVID‐19 outcomes on gynecologic oncology. Outside of the submitted work, RM reports grant funding received from the Eve Appeal, Barts Charity, CRUK, and Rosetrees Trust and speaker/lecture fees from AstraZeneca and GSK. Outside of the submitted work, SG‐M is an officer and trustee for the BGCS and member of the RCOG Academic and eLearning Editorial Boards and CPD Committee. Relating to the submitted work, SS received funding from the BGCS for COVIDSurg‐Gynaecological cancer via the University of Birmingham. SO declares no conflicts of interest.
